# Cytotoxic effect of a combination of bluetongue virus and radiation on prostate cancer

**DOI:** 10.3892/etm.2014.1751

**Published:** 2014-06-02

**Authors:** WEI WANG, MEI-NAN CHEN, KAI CHENG, LI-LI ZHAN, JIE ZHANG

**Affiliations:** 1 Department of Urology, Renmin Hospital of Wuhan University, Wuhan, Hubei 430060, P.R. China; 2 Department of Epidemiology and Biostatistics, School of Public Health, Tongji Medical College, Huazhong University of Science and Technology, Wuhan, Hubei 430030, P.R. China; 3 Huangshi Central Hospital, Hubei Polytechnic University, Huangshi, Hubei 435000, P.R. China; 4 Department of Obstetrics and Gynecology, Renmin Hospital of Wuhan University, Wuhan, Hubei 430060, P.R. China

**Keywords:** bluetongue virus, radiotherapy, prostate cancer, combined effects, cell apoptosis

## Abstract

This study aimed to investigate the lethal effect of the combination of bluetongue virus (BTV) and radiation on RM-1 murine prostate cancer cells *in vitro* and *in vivo*. Various cell lines were infected with BTV and the cytotoxicity was tested by a lactate dehydrogenase (LDH) release bioassay. Additionally, the RM-1 cells were treated with radiation and/or BTV to assess cell viability using the Cell Counting Kit-8 method. The levels of apoptosis of the RM-1 cells were detected by fluorescence-activated cell sorting (FACS). To identify a possible mechanism for the radiation-induced change in the oncolytic activity of BTV, cell cycle analyses were performed. The effects of different schedules of BTV and radiotherapy on cytotoxicity were assessed *in vitro* and the combined effect was also assessed in tumor models *in vivo*. The results demonstrated that BTV had a selective cytotoxic effect on RM-1 and PC-3 cancer cells, but did not affect normal cells, specifically, human umbilical vein endothelial cells and smooth muscle cells. The combination of BTV and radiation enhanced the cytotoxicity compared with that of each agent alone and had a synergistic effect *in vitro* and *in vivo*. The results of the FACS confirmed that radiotherapy induced apoptosis, as did BTV alone, and the combination treatment generated the most prominent levels of apoptosis, which were the highest in the early stage. The analysis of the cell cycle indicated that the G2-M phase levels increased after irradiation followed by infection with BTV. In conclusion, the combination of BTV and radiotherapy had an enhanced cytotoxic effect on RM-1 cells *in vitro* and *in vivo* compared with that of either treatment alone, and demonstrated a synergistic efficacy, in addition to a marked apoptosis-inducing effect. These results support the future investigation of BTV for potential clinical use in patients with prostate cancer.

## Introduction

Prostate cancer is a common type of tumor in elderly males, and is the second most common cancer in males worldwide (1). Prostate cancer accounts for 11% of all types of tumor in males, and ~9% of the total mortalities in males with cancer (2). The therapy of prostate cancer is interlinked with clinical staging, mainly comprising active surveillance, radical surgery, radiotherapy and endocrine therapy. As the early stage of prostate cancer is always accompanied by a lack of clinical symptoms, the majority of patients miss out on the opportunity of surgical treatment due to the detection of their prostate cancer at the advanced stage, which leads to a poor prognosis. Therefore, it is necessary to explore novel anticancer drugs.

As a novel biological treatment strategy, oncolytic viruses have become a topic of particular interest in the laboratory and they are specifically targeted to kill tumor cells while leaving normal cells intact (3). Through intensive investigation, the oncolytic potentials of viruses have been discovered in six major viral families: reovirus type 3, flaviviruses, papillomaviruses, hepadnaviruses, retroviruses and herpesviruses (4,5). Among these, reovirus and smallpox virus are in phase II clinical trials at present (6,7). However, as the majority of current oncolytic viruses undergoing study are modified viruses, the exploration of novel natural oncolytic viruses with little toxicity is urgently required. A study has revealed that reoviruses preferentially replicate in cells that have an activated Ras pathway due to either Ras mutation or upregulated levels of epidermal growth factor receptor signaling (8). As Ras activation exists in 30–40% of human tumors but not in normal cells (9), the use of oncolytic viruses presents an attractive prospect. Bluetongue virus (BTV) is of the *Orbivirus* (ring or circle in Greek) genus in the Reoviridae family, which also includes reoviruses (10). BTV is the causal agent of bluetongue disease in domestic cattle and wild ruminants and is non-pathogenic to humans; thus, humans do not have pre-existing antibodies to BTV (11). BTV is a natural oncolytic virus that has been confirmed to exert powerful oncolytic activity against numerous types of cancer cell (12). A study has shown that reoviruses exert effective cytotoxicity in prostate cancer (13).

Radiation therapy is a frequently used treatment for prostate adenocarcinoma. However, despite significant improvements in delivery technologies, numerous patients develop recurrence following treatment with curative intent. As prostate cancer progresses, the current therapeutic options for advanced prostate cancer are limited to androgen deprivation and/or the cytotoxic effects of high-dose radiation on the surrounding tissues with the aim to extend the survival time of the patient while maintaining quality of life (14). Therefore, it may be hypothesized that the combination of radiation with BTV to target prostate cells represents an attractive treatment option. In the present study, the RM-1 mouse prostate cancer cell line was used to investigate the effect of BTV in combination with radiation.

## Materials and methods

### Cell lines

BTV was preserved by our own lab (Central Laboratory of Renmin Hospital Of Wuhan University, Wuhan, China). RM-1 (mouse prostate cancer cell line) and PC-3 (human prostate cancer cell line) cells were obtained from the China Center for Type Culture Collection (Wuhan, China). Vero (African green monkey kidney cell line) cells were preserved by our lab and used for replication of BTV. Human umbilical vein endothelial cells (HUVECs) were provided by Professor Jing-Yue Hu of the Central Laboratory of Renmin Hospital of Wuhan University (Wuhan, China). Smooth muscle cells (SMCs) were separated from rat aortic smooth muscle and maintained by Dr Zong-li Ren (Department of Cardiothoracic, Renmin Hospital Of Wuhan University). All cell lines, with the exception of Vero, were cultured in RPMI-1640 medium (Gibco-BRL, Carlsbad, CA, USA) containing 10% (v/v) fetal bovine serum (FBS; HyClone Laboratories, Inc., Logan, UT, USA) and 1% (v/v) penicillin/streptomycin. The Vero cells were maintained in Dulbecco’s Modified Eagle’s Medium (DMEM; Gibco-BRL) supplemented with 10% (v/v) FBS and 1% (v/v) penicillin/streptomycin. The cell lines were maintained at 37°C in an atmosphere containing 5% CO_2_. Irradiation and BTV infection was conducted in DMEM containing 2% (v/v) FBS and 1% (v/v) penicillin/streptomycin.

### Replication of BTV and infection

The BTV stock was initially diluted to the highest multiplicity of infection (MOI) that was to be used in the experiment (100 MOI) and subsequently serially diluted to the various MOIs required for each individual experiment. BTV-sensitive Vero cells were used for replication of the BTV. The Vero cells were cultured in the DMEM medium with high sugar containing 10% FBS in 25-ml culture flasks. The cells were grown until they covered 70% of the bottom of the flask, and then the culture medium was discarded and the cells were inoculated with 1 ml purified virus suspension. Subsequently, the cells were incubated with the virus for 1–2 h at 37°C and 5% CO_2_, after which time the medium was aspirated and replaced with fresh medium containing 2% FBS. The cells were observed every 6 h and by the time the cytopathic effect (CPE) reached 90%, the cells were frozen and thawed three times, and then centrifuged at 4°C and 1,600 × g for 15 min. The lysate was collected in 1-ml tubes and maintained at −80°C.

### TCID_50_ assays

Vero cells were plated at a density of 1×10^4^ cells per well in a 96-well plate. The viral suspension was sequentially diluted in 10-fold series, from 10^−3^ to 10^−8^. When the cells attached to the bottom of the plates, various MOIs of 100 μl BTV were added to each well (each MOI was applied to eight parallel wells). To the control cells were added 100 μl growth liquid and 100 μl cell suspension. Following incubation at 37°C for 3 h, the lysates or viral suspensions were removed and replaced with DMEM containing 2% (v/v) FCS and 1% (v/v) glutamine, and the cells were then incubated for a further 6–7 days at 37°C. The change in the CPE of each well was observed every 12 h, and the viral titer was calculated using the Karber statistical method when the results were steady.

### Targeting character of BTV

RM-1 and PC-3 cells, HUEVCs and SMCs were plated in six-well plates at a density of 1×10^6^ cells per well individually. When the cells covered 70% of the bottom of the culture flasks, the cells were infected with BTV at a MOI of 1.0 for 2 h and then the medium was replaced with fresh medium containing 2% (v/v) FBS and 1% (v/v) penicillin/streptomycin. The corresponding mock-infected cultures were subjected to the same procedure using virus-free phosphate-buffered saline (PBS) instead of the viral suspensions. Changes in the cells were observed every 8 h and then images were captured using a microscope (Olympus, Tokyo, Japan). After 24 h, cell survival was assessed by a lactate dehydrogenase (LDH) release bioassay. The LDH release assay was a colorimetric assay for the quantification of cell death and cell lysis. The LDH assay was performed using LDH Cytotoxicity Assay kit (cat. no. C0016, Beyotime, Haimen, China). After cells infected by BTV 24 h later, 200 ml LDH release reagent was added to each well in six-well plates to incubate for 45 min. Afterwards, the cell culture plate was centrifuged in microplate-centrifuge at 400 × g for 5 min, and the 120 μl supernatant of each group was added to 96-wells plate respectively. The absorbance was read at 492 nm by the microplate spectrophotometer (Perkin Elmer, Akron, OH, USA). Cell survival= (2-Abs_treated cells_/Abs_untreated cells_) ×100%.

### Cytotoxic activity of the combination of irradiation and BTV infection

RM-1 cells were plated in 96-well flat-bottom plates in 1 ml media. The cells were treated with the media alone (control wells), radiation alone, BTV alone or combination therapy using radiation therapy and BTV. The BTV infection was conducted at MOIs of 10^−3^, 10^−2^, 10^−1^, 1, 10 and 100 in a total volume of 100 μl medium. The radiotherapy was performed using serial dilutions of radiation (2, 4, 5, 6, 8 and 10 Gy). To assess the combined effect of the two agents, 5 Gy of radiation was used and cells were infected with various MOIs. The effect of the sequence of the combined treatment was evaluated via two protocols. In the first schedule, the RM-1 cells were infected with BTV at 0.1 MOI and radiation doses of 0, 4 and 5 Gy were administered 24 h later. In the second schedule, the order of treatments was reversed such that the same radiation doses (0, 4 and 5 Gy) were delivered 24 h prior to infection of the cells with BTV at 0.1 MOI. The percentage survival for each group was determined on each day 24 h after treatment by the Cell Counting Kit-8 (CCK-8) method and the absorbance (A) was measured at 450 nm. The cell survival rate was calculated using the following formula: Cell survival rate (%) = (A of the experimental group − A of the blank control group)/(A of the control group − A of the blank control group).

### Fluorescence-activated cell sorting (FACS) analysis of cell survival using Annexin V/propidium iodide (PI)

The RM-1 cells were treated with 5 Gy radiation alone, BTV (0.1 MOI) alone, the combination treatment (0.1 MOI BTV 24 h after 5 Gy), or with mock-infection and mock-radiation (control). All cell cultures were harvested 24 h post-infection and the media were collected, then re-suspended at a density of 1×10^6^ cells in 500 μl binding buffer. The cells were stained with 10 μl PI and 5 μl Annexin V-fluorescein isothiocyanate by incubation for 5 min. A total of 10,000 events were collected and the proportion of apoptotic cells was analyzed with a FACSCalibur flow cytometer (Becton-Dickinson, Franklin Lakes, NJ, USA).

### Cell cycle analysis by FACS

The procedure of the cell treatment for the cell cycle analysis was the same as that of the FACS analysis of cell survival. Subsequently, the cells were collected, washed twice with 100 μl PBS and fixed in 70% ice-cold ethanol at 4°C overnight. On the following morning, 5 μl RNase was added to the cells and then they were incubated at 37°C. After 20 mins, the cells underwent further PI staining and the final concentration of PI staining was 40 mg/ml. All samples were analyzed on a FACSCalibur flow cytometer using FlowJo software (TreeStar Inc., Ashland, OR, USA).

### Antitumor effect of BTV and radiation on RM-1 tumor volume in vivo

C57BL6 mice were purchased from the Beijing HFK Bioscience Co., Ltd. (Beijing, China). The care and use of the animals followed the recommendations and guidelines of the National Institutes of Health (IACUC; approval number, 2011006) and they were reviewed and approved by the Institutional Animal Care and Use Committee of Wuhan University. The animals were maintained under specific pathogen-free conditions and fed a strictly sterile diet. In all cases, tumors were established by injection of RM-1 cells suspended in 100 μl PBS in the right flank and the tumor size of all mice was observed every two days. Approximately 12–14 days following the injection, the mice with tumors of 6–8 mm in diameter were selected to be involved in the experiment. In total, 24 mice were allocated to the study and randomly divided into four groups: i) Mock; ii) radiation alone (10 Gy in five fractions); iii) BTV alone [intratumoral injection of 1×10^7^ plaque-forming units (PFU) every other day for seven times]; and iv) BTV plus radiation (10 Gy in five fractions and seven doses of intratumoral BTV, administered as 2-Gy doses of radiation followed by a single intratumoral injection of 1×10^7^ PFU BTV 24 h later). The longest diameter, d1 (mm), and shortest diameter, d2 (mm), of the tumors were measured every other day using Vernier calipers for 20 days. The tumor volume, V, was calculated from the following formula: V = d1 × d2^2^/2. The mice were sacrificed if the largest tumor dimension was >4 cm or there was ulceration of the skin.

### Statistical analysis

Experiments were repeated at least three times and the results are expressed as the mean ± standard deviation. Comparative analyses between groups were performed using post-hoc analysis or Wilcoxon Z test. The software SPSS, version 14.0 (SPSS, Inc., Chicago, IL, USA) was used to perform the statistical analysis and GraphPad Prism, version 5.0 (GraphPad Software, Inc., La Jolla, CA, USA) was used for drawing the figures. The effect of the combined therapy on cell proliferation was measured by calculating the combination index (CI) values using CalcuSyn software (Biosoft, Cambridge, UK). Based on the median-effect principle of Chou and Talalay (15), the CI affords a quantitative measure of the degree of interaction between two or more agents. CI>1 signifies antagonism, CI<1 signifies synergy and CI=1 signifies an additive interaction.

## Results

### BTV targets cancer cells

**Figure 1:**
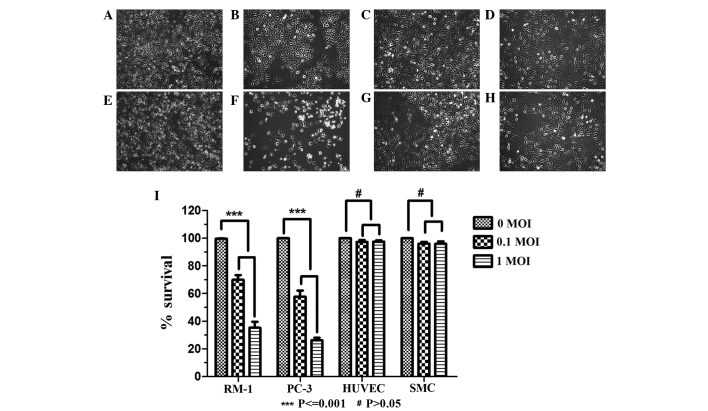
(A–D) Mock infection. (E–H) Different cells infected by BTV at 1.0 MOI. (A and E) RM-1 and (B and F) PC-3 cells; (C and G) HUVECs and (D and H) SMCs. (I) The survival of the various cell types was measured by a LDH release bioassay at 24 h after infection. Data are normalized to the uninfected control (0 MOI). All data are representative of at least six repeats and are normalized to the respective controls (0). MOI, multiplicity of infection; HUVEC, human umbilical vein endothelial cells; SMC, smooth muscle cells; BTV, bluetongue virus; LDH, lactate dehydrogenase.

The effect of BTV infection in a range of cell lines was assessed. RM-1 and PC-3 cells, HUVECs and SMCs were infected with BTV at 0.1 MOI or 1.0 MOI. The results demonstrated that in the RM-1 and PC-3 tumor cells, CPEs were observed and the CPEs reached 90% 48 h later (data not shown). Various pathological changes of the cells occurred and included the following: Intracellular appearance of a large number of particles, the rounding and reduction in size of a number of cells, expansion of the intervals among cells, enhancement of the cell contours and loss of inherent morphological characteristics under normal conditions. A large amount of exfoliated cells and cell debris floating in the medium were observed (Fig. 1A–H). In the normal HUVEC and SMC groups, CPEs were not observed in the treatment group, with no difference in appearance compared with that of the control group. Subsequently, a LDH release bioassay was used to detect cell survival following infection with BTV. The results showed that BTV had a significant inhibitory effect on the RM-1 and PC-3 tumor cells (P<0.001), and no effect on the normal HUVECs and SMCs (P>0.05) compared with the survival of the untreated cells (Fig. 1I).

### Combination of irradiation and BTV infection

**Figure 2:**
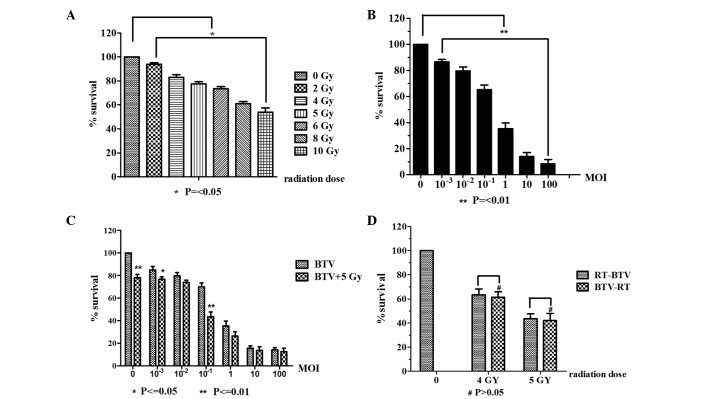
(A) RM-1 cells were exposed to different doses of radiation and 24 h later cell survival was measured by CCK-8, by which it was observed that that the RM-1 cells were moderately susceptible to radiation therapy of 5 Gy. (B) Various dilutions of BTV exert an antitumor effect against RM-1 cells. The cells were infected with BTV from 10^−3^ to 100 MOI and the viability was assessed by CCK-8 24 h post-infection. (C) RM-1 cells were plated at 10,000 cells/well, irradiated with 5 Gy, and infected with BTV at a range of MOIs 24 h post-radiation therapy. Cell survival was tested by CCK-8 24 h post-infection. (D) RM-1 cells were irradiated with 4 or 5 Gy and 24 h later were infected with BTV at 0.1 MOI (IR→BTV), or cells were infected with BTV at MOI of 0.1 and exposed to 4 or 5 Gy radiation 24 h post-infection (BTV→IR). Cell viability was tested by CCK-8 24 h after the final treatment. All data are normalized to the control group (0) and data are representative of at least six repeats. MOI, multiplicity of infection; BTV, bluetongue virus; RT, radiation therapy; CCK-8, Cell Counting Kit-8.

In the initial experiments, the cytotoxicity of radiotherapy or BTV infection alone was tested prior to examining the combination treatment using CCK-8. As shown in Fig. 2A, the cells received 2, 4, 5, 6, 8 or 10 Gy of radiation and 24 h later, the cell survival was measured. The control group received mock irradiation. As shown in Fig. 2A, it was observed that as the dose of radiation increased, the inhibitory effect was increasing evident. When the radiation dose was 10 Gy, the survival rate of the cells was 53.75±7.32%. Statistically significant differences existed between the control and irradiated groups. Prominent BTV MOI-dependent cytotoxicity is shown in Fig. 2B. These data indicate that RM-1 cells have a high susceptibility to BTV infection. The effect of BTV was most marked at the highest MOI (P<0.001).

In Fig. 2C, the results indicated markedly enhanced cytotoxicity of the combination of BTV and radiation compared with that of BTV alone. The effect of the combination treatment was superior to that of various dilutions of BTV or 5 Gy radiation alone (P<0.001), and the combined effect was particularly evident at the lower MOIs (MOI<1) and most marked at the MOI of 0.1 (P<0.001). By contrast, the effect of the combined treatment was less evident compared with that of BTV alone when BTV was used at the high MOIs (>10).

To evaluate the effects of the radiation schedule on the cytotoxicity of BTV, a series of experiments was conducted in which the interval between the two treatments was varied. When the sequence of treatments was reversed and the BTV infection was performed 24 h prior to irradiation with 4 and 5 Gy, no evident difference was identified in comparison with BTV infection following radiation for 4 Gy (63.4±11.8 vs. 61.4±11.3%; P>0.05) and 5 Gy (44±10.3 vs. 42±15.0%; P>0.05; Fig. 2D).

For the combination treatment, analysis of the interaction between radiation and BTV was conducted based on the principle of Chou and Talalay. The CIs were computed and are shown in Table I. Using this methodology, a CI<1.0 is indicative of synergy, with a CI>1.0 denoting antagonism and CI=1 indicating an additive effect. From the results, synergism (CI<1.0) was observed for the RM-1 cells exposed to 5 Gy radiation combined with BTV at MOI>0.03, and CI>1.0 was only observed when the BTV was at a low MOI. The susceptibility of the RM-1 cells to radiation may have affected the results.

### BTV and radiation individually induce cell cycle arrest and together increase the levels of apoptosis

**Figure 3:**
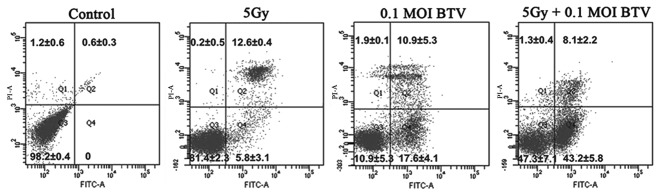
RM-1 cell survival was assessed by FACS with PI/Annexin-V staining. The cells were treated with radiation alone (5 Gy), BTV alone (0.1 MOI) or combined therapy (5 Gy + 0.1 MOI). The data present the survival of cells (%). Data are representative of three similar experiments. FITC, fluorescein isothiocyanate; MOI, multiplicity of infection; BTV, bluetongue virus; FACS, fluorescence-activated cell sorting; PI, propidium iodide.

The apoptosis-inducing effect of either irradiation or BTV on RM-1 cells was detected by FACS analysis of the cell survival using PI/Annexin-V staining. As shown in Fig. 3, there was no evident apoptosis in the control group. Radiotherapy mainly lead to apoptosis at the late stage (12.6±0.4%) whereas infection with BTV induced equal levels of apoptosis at the early (17.6±4.1%) and late (10.9±5.3%) stages. By contrast, the combination of BTV and radiation generated the most prominent levels of apoptosis (51.3±3.2%) and the levels of apoptosis at the early stage (43.2±5.8%) were the highest.

### BTV and/or radiation induce changes in the cell cycle

**Figure 4:**
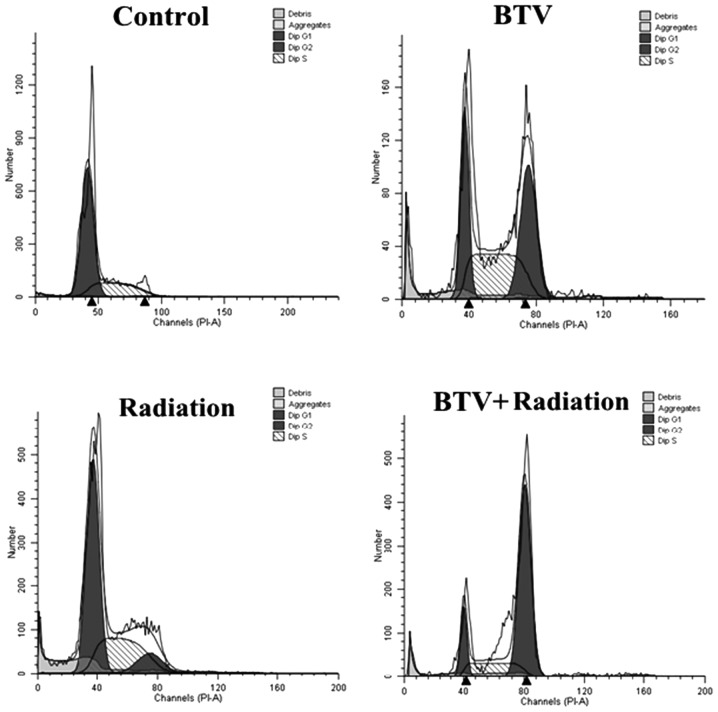
Effects of different treatment methods on the cell cycle of RM-1 cells. The cells were treated with radiation alone (5 Gy), BTV alone (0.1 MOI) or combined therapy (5 Gy+0.1 MOI) or were mock treated. The data presents the cell cycle of the RM-1 cells. Data are representative of three similar experiments. BTV, bluetongue virus; MOI, multiplicity of infection.

One of the most important potential mechanisms of the radiation-enhanced cytotoxicity of BTV infection may be the blockade of the cell cycle. Treatment with 5 Gy radiation induced an increase in the number of RM-1 cells in the G2-M phase, while infection with BTV resulted in an accumulation of cells in the S and G2-M phases with a marked reduction in the number of cells in G1 phase compared with those of the control group. By comparison, when the RM-1 cells were infected with 1 MOI BTV following treatment with 5 Gy radiation, the number of cells in the G2-M phase increased the most compared with that in the other groups (Table II; Fig. 4). This revealed that the combined treatment induces increased levels of apoptosis compared with those of either treatment alone.

### Combined BTV and radiation treatment enhances the delay of tumor growth in vivo in C57BL6 mice

**Figure 5:**
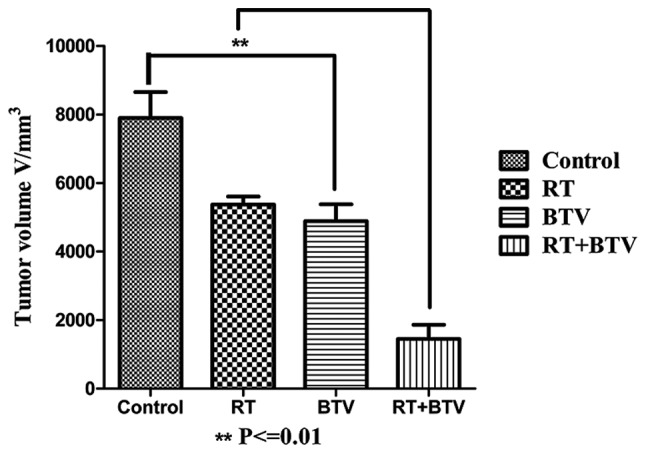
Tumors grown in C57BL6 mice that were divided into four groups: Control, mock radiation therapy and mock infection; radiation therapy (10 Gy in five fractions); BTV (intratumoral injection of BTV at 1×10^7^ PFU seven times); combined radiation therapy and BTV. The results present the average of the sample group (n=6 per group) and are presented with the standard errors of the mean. RT, radiation therapy; BTV, bluetongue virus; PFU, plaque-forming units.

Following establishment of tumor-burdened mice for 7–10 days, 4–5 mm bumps were identified in the subcutaneous tissue and they gradually increased in size. All mice tolerated the radiation and no evident reduction in the physical quality and mortality rate of the mice was observed in the study. The tumors in the control group grew the most rapidly and in the other three groups, the tumors grew slower than those of the control group. Among them, the growth of the tumors in the combined group was the slowest, and there were statistical differences compared with that of the other three groups. Twenty days after treatment, the mean tumor volume of the mice which received combination therapy was reduced ~4-fold (compared with that of the control), 2-fold (compared with that of radiation alone) and 1.9-fold (compared with that of BTV alone) (P<0.01; Fig. 5). There was no evidence of exacerbation of the cutaneous toxicity in the mice treated with the combination therapy compared with that of radiotherapy alone.

## Discussion

The combination of oncolytic viruses and radiotherapy presents an emerging and promising novel approach for the treatment of various types of malignant tumor. Numerous studies have confirmed that combination therapies have shown synergistic antitumor effects in preclinical models (16–19), but the majority of them have used a gene-modified virus. BTV is a type of natural targeted antitumor oncolytic virus that easily multiplies and costs less compared with genetically modified viruses. Radiation therapy is a standard treatment for patients with prostate cancer, but long-term or high-dose rate radiation inevitably induces many more side-effects. Therefore, combined therapy may enable the dose of radiation to be reduced and thus decrease the side-effects, or improve the efficiency of an equal dose of radiation.

In the present study, different types of cell infected by BTV were examined, and the evidence demonstrated that BTV selectively infected and was cytotoxic to RM-1 and PC-3 cancer cells but not cultured normal primary cells. This is significant in clinical therapy as no normal organs should be infected by BTV and thus side-effects are not likely to occur in other systems.

The present study also demonstrated that BTV exhibited a dose-dependent cytotoxicity to the RM-1 cells, as did the radiation. When BTV and radiation were combined together, the results showed a marked increase in the cytotoxicity compared with that of each treatment alone *in vitro*, particularly at moderate input MOIs of BTV. Furthermore, CIs were calculated to examine the synergistic effect of BTV and radiation (CI<1). In addition, the enhanced cytotoxicity was not associated with the sequence of administration of the two agents. This may be of relevance to clinical studies that use fractionated radiotherapy schedules as it is likely that, if multiple viruses are used, BTV administration will follow some fractions of radiation but precede others. The data from the *in vivo* experiment in the present study further confirmed that the combination of BTV and radiotherapy notably increased the cytotoxicity compared with that of each agent used alone, particularly at moderate MOIs of BTV. Also, it was demonstrated that BTV is stable in irradiated tissues and acts independently of the treatment schedule.

In agreement with our assumptions, the results of the apoptosis analysis by FACS using PI/Annexin-V dye in the present study indicate that the increased cytotoxicity may be due to a notable increase in apoptosis caused by the combined treatment. Cell cycle analysis for the combined therapy showed that the G2-M phase dominates the cell cycle following the combination treatment, and this may result in the arrest of RM-1 cells in the G2/M phase and ultimately an antitumor effect.

In conclusion, the results of the present study demonstrate that the combined treatment exerts a synergistic antitumor effect on RM-1 cells *in vivo* and *in vitro*. These data support the future clinical investigation of the use of BTV combined with radiation for the therapy of prostate cancer with the purpose of reducing toxicity while increasing efficacy.

## Figures and Tables

**Table I:** Required doses of radiation and virus to achieve various FA levels of RM-1 cells.

FA	RT (Gy)	BTV (MOI)	5Gy RT + BTV (MOI)	CI
LD_30_	6.40 (5.93,6.91)	0.03 (0.00,0.86)	0.00 (0.00,0.10)	1.11 (1.07,1.17)
LD_40_	8.44 (7.79,9.14)	0.10 (0.00,2.64)	0.02 (0.00,0.58)	0.94 (0.89,1.00)
LD_50_	10.86 (9.99,11.83)	0.29 (0.01,7.74)	0.07 (0.00,1.85)	0.83 (0.76,0.90)
LD_60_	13.99 (12.75,15.35)	0.88 (0.03,23.50)	0.26 (0.01,6.17)	0.75 (0.67,0.84)
LD_70_	18.43 (16.64,20.41)	2.90 (0.10,82.50)	0.99 (0.04,24.24)	0.70 (0.61,0.81)
LD_80_	25.79 (22.98,28.94)	12.46 (0.39,403.55)	5.13 (0.19,138.61)	0.69 (0.58,0.82)
LD_90_	42.75 (37.26,49.06)	111.51 (2.55,4873.04)	60.98 (1.70,2191.79)	0.73 (0.60,0.89)

LDx, lethal dose x, dose required to kill x% of cells. FA, fraction affected; RT, radiation therapy; BTV, bluetongue virus; MOI, multiplicity of infection; CI, combination index.

**Table II:** Cell cycle distribution (%) induced by BTV and/or radiation.

Treatment	G0/G1	S	G2/M
Control	72.70±3.10	27.10±5.50	0.29±7.30
BTV	25.92±4.90	38.03±1.20	36.04±9.00
Radiation	58.55±2.80	32.91±10.30	10.54±1.00
BTV + radiation	6.46±3.20	32.55±4.40	60.99±8.30

BTV, bluetongue virus.
